# Comparative Study of Anti-Gouty Arthritis Effects of Sam-Myo-Whan according to Extraction Solvents

**DOI:** 10.3390/plants10020278

**Published:** 2021-02-01

**Authors:** Yun Mi Lee, Eunjung Son, Dong-Seon Kim

**Affiliations:** Herbal Medicine Research Division, Korea Institute of Oriental Medicine, 1672 Yuseong-daero, Yuseong-gu, Daejeon 34054, Korea; candykong@kiom.re.kr (Y.M.L.); ejson@kiom.re.kr (E.S.)

**Keywords:** Sam-Myo-Whan, traditional medicine, gouty arthritis, inflammation, monosodium urate

## Abstract

Sam-Myo-Whan (SMW) has been used in Korean and Chinese traditional medicine to help treat gout, by reducing swelling and inflammation and relieving pain. This study compared the effects of SMW extracted by using different solvents, water (SMWW) and 30% EtOH (SMWE), in the treatment of gouty arthritis. To this end, we analyzed the main components of SMWW and SMWE, using high-performance liquid chromatography (HPLC). Anti-hyperuricemic activity was evaluated by measuring serum uric acid levels in hyperuricemic rats. The effects of SMWW and SMWE on swelling, pain, and inflammation in gouty arthritis were investigated by measuring affected limb swelling and weight-bearing, as well as by enzyme-linked immunosorbent assays, to assess the levels of proinflammatory cytokines and myeloperoxidase (MPO). In potassium oxonate (PO)-induced hyperuricemic rats, SMWW and SMWE both significantly decreased serum uric acid to similar levels. In monosodium urate (MSU)-induced gouty arthritis mice, SMWE more efficiently decreased paw swelling and attenuated joint pain compare to SMWW. Moreover, SMWE and SMWW suppressed the level of inflammation by downregulating proinflammatory cytokines (interleukin-1*β*, tumor necrosis factor-*α*, and interleukin-6) and MPO activity. HPLC analysis further revealed that berberine represented one of the major active ingredients demonstrating the greatest change in concentration between SMWW and SMWE. Our data demonstrate that SMWE retains a more effective therapeutic concentration compared to SMWW, in a mouse model of gouty arthritis.

## 1. Introduction

Gout is a metabolic disease caused by increased blood uric acid levels (hyperuricemia) and the deposition of monosodium urate (MSU) crystals in the joints, bone, and subcutaneous tissues. Moreover, gout is closely associated with chronic hyperuricemia, which can markedly reduce patient quality of life due to the severe associated pain [1,2]. Currently, a number of anti-gout agents, including anti-inflammatory drugs (colchicine and indomethacin) as well as urate-lowering drugs (allopurinol and benzbromarone) are often selected as primary therapies for gout. Although these agents are generally effective, they are also associated with various adverse effects, including gastrointestinal, hepatic, and renal toxicity and hypersensitivity [3]. Therefore, it is critical to develop novel agents with fewer associated adverse effects while retaining, or improving, their clinical efficacy. Existing evidence suggests that several natural agents exhibit beneficial efficacy and produce fewer side effects in the treatment of gouty arthritis [4,5]. We have, therefore, focused our research on these candidate natural products.

Sam-Myo-Whan (SMW) has been a common prescription for the treatment of gout and is recorded in traditional Eastern medicine, such as Donguibogam and Chinese Pharmacopoeia. It has good therapeutic efficacy in reducing dampness (edema), decreasing heat and swelling (inflammation), and alleviating pain [6,7]. Moreover, SMW and modified SMW, which is combined with other herbal medicines, are commonly used clinically for the treatment of gouty and rheumatoid arthritis in China [8,9]. SMW is composed of Phellodendri cortex (*Phellodendron chinense* Schneider)**,** Atractylodes rhizome (the rhizome of *Atractylodes chinensis* Koidzumi), and Achyranthes radix (the root of *Achyranthes japonica* (Miq.) Nakai) in a compatible ratio of 2:3:1. SMW has been shown to inhibit lipopolysaccharide (LPS)-induced inflammatory responses by reducing nitric oxide (NO), tumor necrosis factor-α (TNF-α) production, and inducible nitric oxide synthase (iNOS) expression in RAW264.7 cells and BV2 cells [7]. SMW produced dual hyperuricemic actions by downregulating hepatic XOD to reduce uric acid production and inhibiting renal mURAT1, to decrease urate reabsorption and enhance urate excretion in hyperuricemic mice [10]. In addition, SMW effectively treats osteoarthritis by suppressing chondrocyte apoptosis, cartilage matrix degradation, and the inflammatory response [11]. SMW also modifies the expression of matrix metalloproteinases (MMPs)-3 and aggrecanases (ADAMTSs)-4, which are considered key enzymes in cartilage matrix degradation, and enhances the expression of gouty arthritis-reduced tissue inhibitors of metalloproteinases (TIMPs)-1 and -3, resulting in the effective inhibition of cartilage matrix degradation in gouty arthritis [12]. Several recent studies have also reported that SMW may exhibit therapeutic synergy in gouty arthritis by regulating numerous biological processes and pathways. These include the lipopolysaccharide-mediated signaling pathway, positive regulation of transcription, Toll-like receptor, Janus kinase–signal transducer and activator of transcription (JAK–STAT), nucleotide binding and oligomerization domain (NOD)-like receptor, and mitogen-activated protein kinase (MAPK) signaling pathways [13,14,15]. In addition, SMW used a modified SMW, adding herbal medicines, to maximize the efficacy of patients with gouty arthritis and to alleviate various symptoms of patients with different phases of gouty pathology. Furthermore, modified SMW has exhibited good results on patients with gout characterized by swelling and edema (dampness-heat type in Chinese medicine) and has been shown to inhibit inflammatory factors in the joint fluid of rats with acute gout arthritis [16,17,18,19]. However, according to these previous reports, the SMW was pulverized to a fine powder and suspended in distilled water, or extracted by refluxing with water. While several studies have reported on the efficacy of SMW as a treatment option, there have been no investigations into the differences in composition and efficacy according to the extraction solvent used. Although traditional Chinese and Oriental herbal medicines have used water extracts, ethanol or ethanol/water mixture has recently been introduced as an extraction solvent for pharmaceuticals and dietary supplements. Moreover, the Korea Food and Drug Administration exempts or requires minimum toxicity test data for drug approval of Oriental herbal medicine when using ethanol content up to 30% in mixture with water as an extraction solvent. Thus, this study investigated the differences and changes in the ingredients and efficacy of SMW according to the extraction solvent, namely water (SMWW) and 30% ethanol (SMWE). The quantities of index components and the anti-gouty arthritis activities of two kinds of SMW extract were compared in rat and mouse models.

## 2. Results

### 2.1. Chemical Profiling Analysis of SMWW and SMWE

**Figure 1:**
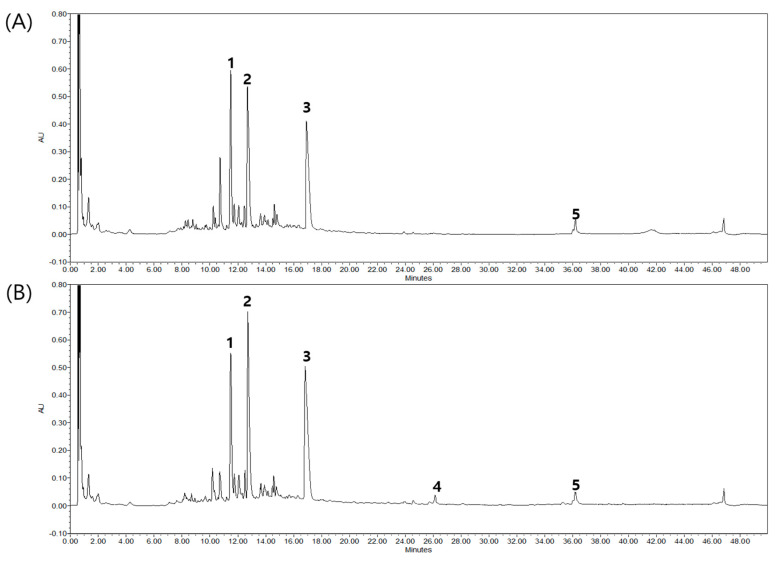
Representative UPLC chromatogram at 200 nm: (**A**) Sam-Myo-Whan (SMW) water extract and (**B**) SMW 30% ethanol extract. (1) Palmatine, (2) armepavine, (3) berberine, (4) atractylenolide III and (5) atractylenolide I.

Based on their UV–Vis absorption spectra and retention times, palmatine, armepavine, and berberine, protoberberine groups with quaternary ammonium salt structures, were identified as major components of SMW. SMWW contained 15.2 ± 0.09 mg/g of palmatine, 18.7 ± 0.17 mg/g of armepavine, and 21.1 ± 0.23 mg/g of berberine; while SMWE contained 14.2 ± 0.40 mg/g of palmatine, 21.2 ± 0.26 mg/g of armepavine, and 27.9 ± 0.16 mg/g of berberine. We also identified small amounts of atractylenolides I and III, which are part of the sesquiterpenoid group with three isoprene units, by comparing their retention times and UV–Vis absorption spectra with their reference standards (Figure 1).

### 2.2. Serum Uric Acid Levels of Hyperuricemic Rats Treated with SMHW or SMHE

**Figure 2:**
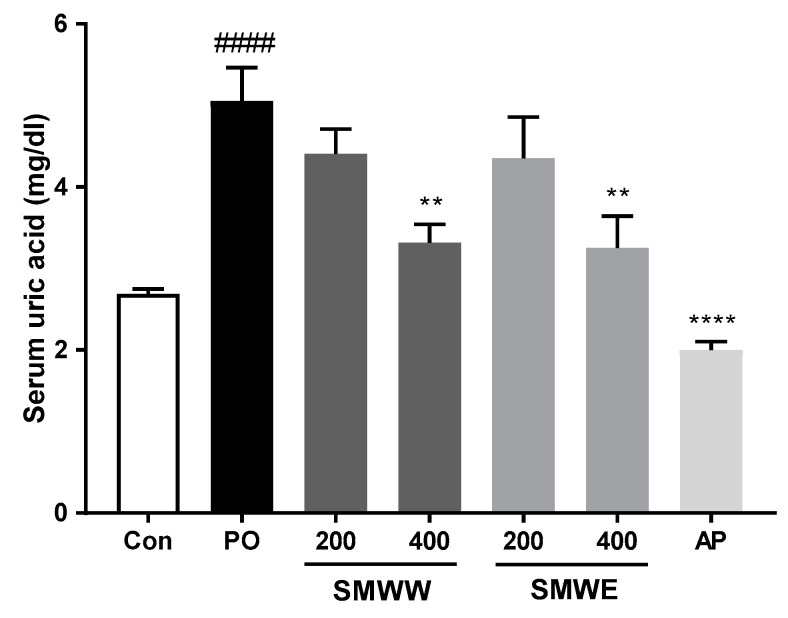
Effects of SMW extracted with water (SMWW) and SMW extracted with 30% EtOH (SMWE) on serum uric acid levels in PO-induced hyperuricemic rats. Con, normal control mice; PO, PO-induced hyperuricemic rat; SMWW, PO rats treated with SMWW; SMWE, PO rats treated with SMWE; AP, PO rats treated with 10 mg/kg of allopurinol. Data are expressed as the mean ± SEM (n = 6). ^####^ *p* < 0.0001 (compared with control group) and ** *p* < 0.01, **** *p* < 0.0001 (compared with PO group).

The effects of SMWW and SMWE on serum uric acid levels in potassium oxonate (PO)-induced hyperuricemic rats are shown in Figure 2. Serum uric acid levels in the PO group rats were significantly increased, compared to those in the Con group (*p* < 0.0001). Treatment with SMWW or SMWE at a 400 mg/kg dose significantly reduced serum uric acid levels by 34.3% and 35.6%, respectively, compared with the PO group (both *p* < 0.01); however, there was no significant difference in efficacy between the two extracts. Rats treated with allopurinol (10 mg/kg) as a positive control showed a 60.4% decrease in their serum uric acid levels (*p* < 0.0001). 

### 2.3. Anti-Inflammatory Effects of SMWW and SMWE on Paw Swelling in MSU-Induced Gouty Arthritis

**Figure 3:**
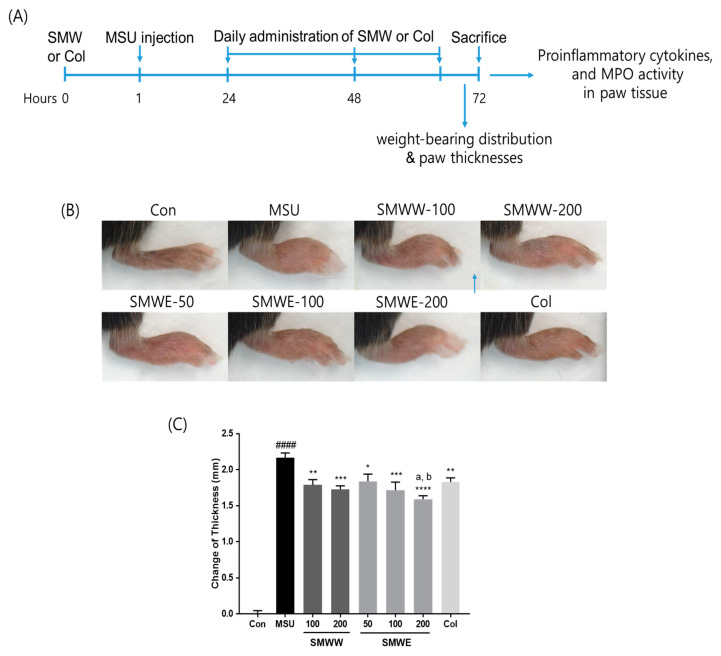
Effect of SMWW and SMWE on paw swelling in mice with monosodium urate (MSU)-crystal-induced gouty arthritis. Con, normal control mice; MSU, MSU-crystal-injected mice; SMWW, MSU mice treated with SMWW; SMWE, MSU mice treated with SMWE; Col, MSU mice treated with 1 mg/kg of colchicine. (**A**) Experimental design. (**B**) Representative images of the right leg from mice in each group. (**C**) Quantification of changes in the thickness of each mouse paw recorded 3 days after the induction of MSU. Data are presented as the mean ± SEM (*n* = 5). ^####^ *p* < 0.0001 (compared with control group); * *p* < 0.05, ** *p* < 0.01, *** *p* < 0.001, **** *p* < 0.0001 (compared with MSU group); ^a^ *p* < 0.05 (compared with 200 mg/kg of SMWW group); ^b^ *p* < 0.01 (compared with 1 mg/kg of colchicine group).

MSU crystals led to a significant increase in paw thicknesses of injected mice compared with the controls (Figure 3B,C). Meanwhile, treatment with SMWW (100 and 200 mg/kg) or SMWE (50, 100, and 200 mg/kg) significantly suppressed MSU-induced paw swelling compared with the MSU group. At the same dose (200 mg/kg), SMWE caused a greater decrease in paw thickness than SMWW, while the 100 mg/kg SMWE dose showed similar anti-inflammatory effects on paw swelling as the 200 mg/kg SMWW dose.

### 2.4. Effect of SMWW and SMWE on Hind Paw Weight-Bearing Distribution 

**Figure 4:**
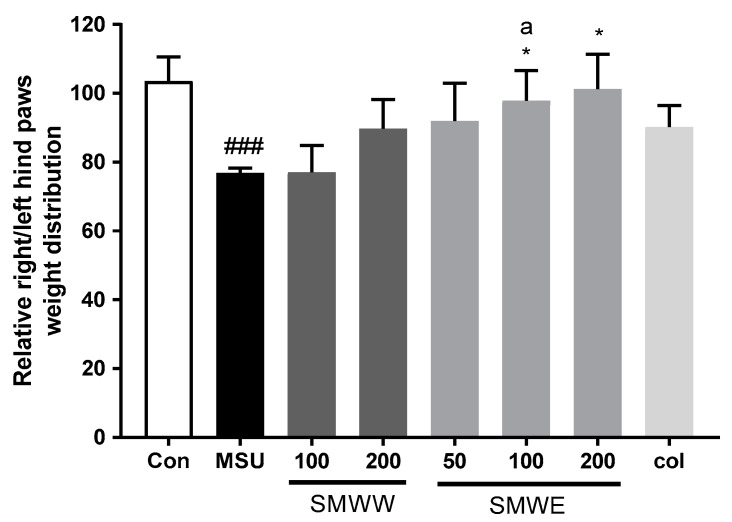
Effect of SMWW and SMWE on hind-paw weight-bearing distribution in mice with MSU-crystal-induced gouty arthritis. The relative right/left hind paw weight-bearing distribution was measured by using a dynamic weight-bearing (DWB) device, compared to that of the MSU-crystal-injected group. Con, normal control mice; MSU, MSU-crystal-injected mice; SMWW, MSU mice treated with SMWW; SMWE, MSU mice treated with SMWE; Col, MSU mice treated with 1 mg/kg of colchicine. Data are presented as the mean ± SEM (*n* = 5). ^###^ *p* < 0.001 (compared with control group); * *p* < 0.05 (compared with MSU group); ^a^ *p* < 0.05 (compared with 100 mg/kg of SMWW group).

In the weight-bearing test, indicating the progressive pain of gouty arthritis, the mice injected with MSU exhibited a clear reduction in weight-bearing on the affected paw, as compared with the control mice (Figure 4). Although hind-paw weight distribution showed no change with a 100 mg/kg SMWW treatment dose, the 200 mg/kg SMWW dose and 50 mg/kg SMWE dose, increased weight distribution to levels similar to the Col treatment group. In particular, the 100 and 200 mg/kg SMWE doses significantly elevated hind-paw weight distribution. 

### 2.5. Effects of SMWW and SMWE on Proinflammatory Cytokines

**Figure 5:**
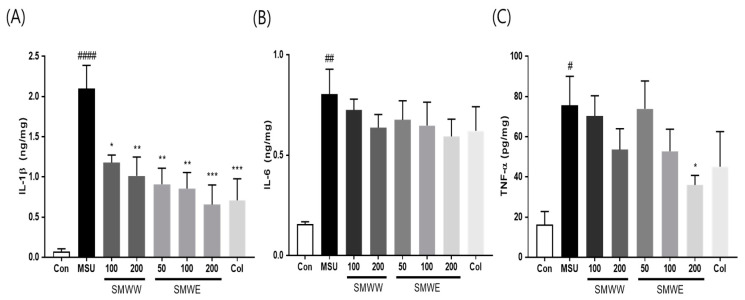
Effects of SMWW and SMWE on proinflammatory cytokines expression in MSU-crystal-injected paw tissue. Con, normal control mice; MSU, MSU-crystal-injected mice; SMWW, MSU mice treated with SMWW; SMWE, MSU mice treated with SMWE; Col, MSU mice treated with 1 mg/kg of colchicine. (**A**) IL-1β, (**B**) IL-6, and (**C**) TNF-α levels measured by ELISA. Data are presented as the mean ± SEM (*n* = 5). ^#^ *p* < 0.05, ^##^ *p* < 0.01, ^####^ *p* < 0.0001 (compared with control group); and * *p* < 0.05, ** *p* < 0.01. *** *p* < 0.001 (compared with MSU group).

We investigated the anti-inflammatory effects of MSU-injection by assessing the levels of IL-1β, IL-6, and TNF-α, using ELISA. The results showed that MSU-injected mice had significantly elevated IL-1β, IL-6, and TNF-α levels (Figure 5). However, SMWW and SMWE treatment significantly downregulated IL-1β production by at least 43.9%, at all treatment concentrations, with the 200 mg/kg SMWE dose displaying the greatest efficacy (68.7% reduction), compared with the Col positive control (66.2% reduction). In addition, the 200 mg/kg SMWE dose effectively reduced TNF-α levels by 52%, while the 200 mg/kg SMWW dose and the 100 mg/kg SMWE dose reduced TNF-α to similar levels (29.2% and 30.3%, respectively). Both SMW extracts exhibited a weak dose-dependent decrease in IL-6 production, however, these results were not statistically significant.

### 2.6. Effects of SMWW and SMWE on MPO Activity

**Figure 6:**
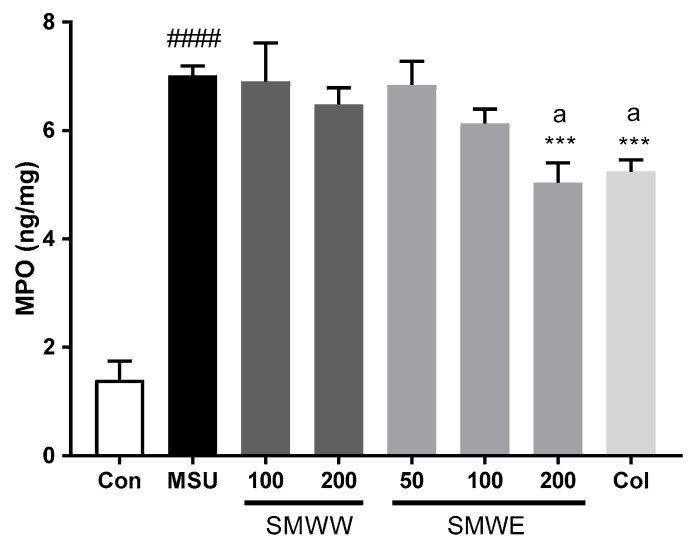
Effects of SMWW and SMWE on myeloperoxidase (MPO) activity in MSU-crystal-injected paw tissue. Con, normal control mice; MSU, MSU-crystal-injected mice; SMWW, MSU mice treated with SMWW; SMWE, MSU mice treated with SMWE; Col, MSU mice treated with 1 mg/kg of colchicine. Data are presented as mean ± SEM (*n* = 5). ^####^ *p* < 0.0001 (compared with control group); *** *p* < 0.001(compared with the MSU group); ^a^ *p* < 0.01 (compared with 200 mg/kg of SMWW group).

To evaluate the possible cellular infiltration induced by MSU, MPO activity was used as an index of neutrophil accumulation. As shown in Figure 6, MSU injection was found to markedly increase MPO activity in affected paw tissue, compared to the controls (*p* < 0.0001). Meanwhile, SMWW and SMWE both reduced MPO activity, with the highest effect observed following administration of SMWE at a dose of 200 mg/kg (*p* < 0.01). The positive control group, treated with 1 mg/kg Col (which inhibits neutrophil recruitment and activation), also exhibited a significant reduction (*p* < 0.05) in MPO levels, compared to the MSU group. 

## 3. Discussion

Gout is a common disease characterized by the deposition of MSU crystals in the joints or subcutaneous tissues, causing acute inflammatory flares or chronic arthritis [20]. Hyperuricemia (high blood uric acid concentration) occurs above the saturation point of MSU, at which point the risk of crystallization increases [21]. MSU crystals result in acute gout attacks characterized by IL-1β-driven acute inflammation, fever, and intense pain caused by neutrophil accumulation and activation in joints. [22]. Therefore, control of hyperuricemia and treatment that reduces inflammation represent the major therapeutic approaches against gouty arthritis [23]. In the present study, we compared the compositional changes as well as treatment efficacy of SMW extracted with water or 30% ethanol. The anti-hyperuricemic effects of SMWW and SMWE in the hyperuricemic animal model, in which serum uric acid levels were increased by intraperitoneal PO injection (to induce hyperuricemia), and the anti-gouty arthritis effects of SMWW and SMWE, were assessed in a gouty arthritis model induced by MSU-crystal injection. In addition, we analyzed the phytochemical contents of SMWW and SMWE, using HPLC. 

The ability of SMW to reduce blood uric acid concentration has been demonstrated previously in many animal experiments and clinical studies [10,24,25], and it was confirmed in our study. Moreover, SMWE and SMWW exhibited similar efficacies. 

The identification of MSU crystals in joint fluid or synovium is the basis for a clinically definitive diagnosis of gout arthritis, as these crystals have been shown to cause strong inflammatory reactions, leading to acute gout arthritis [26,27]. The most significant symptom of gouty arthritis is swelling and pain, which is observed in the mice injected with MSU [26,28]. In the present study, the MSU-injected mice showed a clear increase in swelling, compared with the controls, and markedly reduced weight-bearing on the affected hind paw, indicating pain. Meanwhile, SMWE treatment markedly prevented the MSU-crystal-induced elevation in paw swelling, compared with that of the SMWW or Col groups. Moreover, the 200 mg/kg SMWE dose elicited excellent pain relief, with hind-paw weight-bearing returning to that similar of the Con group. These results demonstrated that SMWE reduced swelling and pain at dosages of 100–200 mg/kg more effectively than did SMWW at 200 mg/kg. 

MSU crystals are one of the most effective proinflammatory stimuli, through their ability to trigger, amplify, and sustain a strong inflammatory reaction in the joint cavity [29]. MSU crystals stimulate the synthesis and release of IL-1β, a key inflammatory cytokine that regulates the differentiation, proliferation, and apoptosis of cells in gout arthritis [30]. In addition, IL-1β induces the expression of a wide range of cytokines, including TNF-α and IL-6, resulting in a large influx of neutrophils into the synovium [31]. In turn, neutrophil interactions with MSU crystals stimulates the synthesis and release of a large variety of pro-inflammatory signals, such as reactive oxygen species, leukotrienes, prostaglandin E2 (PGE2), TNF-α, IL-1, IL-6 and IL-8. This response promotes the vasodilation, erythema and pain associated with acute gout attack [23,32]. Thus, inhibiting MSU-induced recruitment of neutrophils and blocking secretion of inflammatory mediators may prove beneficial for the control and management of acute gouty arthritis [29]. 

Our results further demonstrated that the levels of IL-1β and TNF-α in the paw tissue were significantly increased in response to MSU, however, became markedly downregulated, in a dose-dependent manner, following SMWW or SMWE treatment. Furthermore, MPO activity was significantly elevated in mice with gouty arthritis, compared to the control group (indicating an influx of neutrophils and acute inflammation), while both SMWW and SMWE effectively decreased MPO activity. Again, SMWE treatment resulted in superior inhibition of MPO activity caopared to SMWW, at a level similar to that of the positive control, colchicine, which is a known regulator of neutrophil activity [33]. These results suggest that SMWE relieves acute gout symptoms caused by MSU crystals by inhibiting the major inflammatory cytokines and suppressing MPO activity, which is a key feature in the initiation and progression of gouty arthritis. Furthermore, our data indicates that SMWE treatment is more effective than SMWW.

Extraction solvents have different abilities to solubilize various biologically active compounds, which can have a significant effect on the content and biological activity of the extract [34,35]. Although SMW has long been used to water extract from herbal medicines consisting of a ratio as 2:3:1 (Phellodendri cortex, Atractylodes rhizome and Achyranthes radix), no studies have reported the specific composition of these compounds. For the single medicinal herb, *Atractylodes japonica*, the extract is reported to contain stigmasterol, hinesol, eudesmol, atractylenolides, atractylon, atractylodin, and sitosterol [36], while methanol extract was reported to contain 0.08% hinesol, 0.09% eudesmol, and 0.02% atractylodin [37]. Moreover, Chikusetsusaponin IVa methyl ester, separated from *Achyranthes japonica* 80% methanol extract, reportedly elicits an anti-inflammatory effect, however, no report has been made on quantity [38]. Additionally, *Phellodendron amurense* is reported to contain alkaloids, such as phellodendrine, magnoflorine, tetrahydropalmatine, columbamine, jatrorrhizine, 8-oxyepiberberine, berberine, palmatine, and bis-[4-(dimethylamino)phenyl] methanone [39]. While most studies of such ingredients are conducted by using non-polar extraction solvents (methanol and ethanol) for a single herb, the only traditional method used includes water extraction in a complex of these three herbs (Phellodendri cortex, Atractylodes rhizome and Achyranthes radix). Alternatively, water and ethanol are commonly used as solvents for the extraction of herbs for preparation of traditional decoction, food ingredients, dietary supplements, etc. Thus, we conducted a study using a 30% ethanol extract, which offers the best efficacy in the range of acceptable ethanol concentrations used in traditional methods.

In this study, SMW was extracted with 30% ethanol or water, and the main ingredients were identified as palmatine, armepavine, and berberine. When SMWE was compared to SMWW, the palmatine content was slightly lower and the armepavine content slightly higher than that of SMWW. However, the berberine content of SMWE was 32.2% higher than that of SMWW. Berberine has been reported to possess a wide range of pharmacological activities, including anti-inflammatory, antimicrobial, antioxidant, hypoglycemic, hypolipidemic, and hepatoprotective properties [40]. Additionally, berberine has been shown to downregulate NLR family pyrin domain-containing protein 3 (NLRP3) and IL-1β expression in MSU-crystal-induced inflammation [41]. Other compounds, such as atractylenolide III (a known anti-inflammatory agent), were only detected in SMWE, albeit in small quantities [42]. It has been shown that extraction using an alcohol/water mixture (versus water alone) increases the content of active components that are insoluble in waterwhile also extracting water-soluble active ingredients, thus optimizing the extraction of relatively small amounts of active ingredients present in natural products [34]. Therefore, it is suggested that small amounts of compound, atractylenolide III, and 32.2% increased, berberine, are characteristic components of SMWE and are bioactive compounds that may affect the mouse gouty arthritis model. The compounds may contribute to synergistic or additional effects, and our results suggest that SMWE is more effective in reducing swelling, pain, and inflammation in MSU-induced gouty arthritis mouse model than SMWW. 

## 4. Materials and Methods

### 4.1. Preparation of SMW

The SMW preparation used in this study was purchased from Kwangmyoungdang Pharms (Ulsan, S. Korea). The voucher specimen was deposited at the Korean Herbarium of Standard Herbal Resources of Korea Institute of Oriental Medicine (2-20-0354~2-20-0356, Daejeon, S. Korea). According to Donguibogam, Phellodendri cortex (Phellodendron chinense Schneider) was stir-fried with Makgeolli (1:10, w/v) for 2 h. The Atractylodes rhizome (Atractylodes chinensis Koidzumi) was soaked in rice-washed water for 3 h and then dried. Each sample was ground into a powder. The mixture was prepared with 60 g of Achyranthes radix (Achyranthe japonica Nakai), 180 g of rinsed Atractylodes rhizome, and 120 g of stir-fried Phellodendri cortex, and was extracted with 2 L of water (SMWW) or 30% ethanol (SMWE), for 3 h, by reflux. These extracts were then concentrated under reduced pressure and freeze-dried.

### 4.2. Components Analysis of SMW

Reference standards, palmatine, armepavine, berberine, atractylenolide III, and atractylenolide I, were purchased from Chemfaces (Hubei, China). After confirming compounds by comparing the retention time and absorption profile of the reference material, each component was quantified through the area comparison.

HPLC analysis was performed on an Acquity UPLC system (Waters, MA, USA) equipped with a quaternary pump, auto-sampler, and photodiode array detector with Acquity UPLC® BEH C18, 100 × 2.1 mm, 1.7 μm. A gradient elution with solvent A (0.1% phosphoric acid) and solvent B (acetonitrile), at a flow rate of 0.5 mL/min, was conducted as follows: 0–2 min, 2–2% B; 2–32 min, 2–50% B; 32–42 min, 50–100% B; 42–45 min, 100–100% B; 45–47 min, 100–2% B; and 47–50 min, 2–2% B. The detection wavelength was set to 200 nm. The column temperature was maintained at 40 °C, and the injection volume was 2 µL.

### 4.3. Animals

Male Sprague Dawley (SD) rats (7 weeks) and male C57BL6 mice (7 weeks) were purchased from Orient Bio (Seongnam, Korea) and housed at a temperature of 22 ± 2 °C in a 50 ± 10% humidity-controlled room under a 12 h light/dark cycle. The animals were allowed *ad libitum* access to a laboratory diet and water. At the end point of the experiment, the rats were anesthetized using zoletil and sacrificed by cervical dislocation. No systemic adverse effects were observed following treatment with SMWW or SMWE, in any study group. The experimental design was approved by the Committee on Animal Care of the KIOM (approval No. 20-016), and the study was conducted in accordance with the Guide for the Care and Use of Laboratory Animals published by the US National Institutes of Health (Bethesda, MD, United States). 

### 4.4. Hyperuricemia Induction and Sample Treatment.

The uricase inhibitor PO was injected into rats, to induce hyperuricemia [43]. The rats were divided into the following seven groups (*n* = 6/group): (1) Controls (Con), (2) PO-treated controls, (3) PO+200 mg/kg SMWW, (4) PO+400 mg/kg SMWW, (5) PO+200 mg/kg SMWE, (6) PO+400 mg/kg SMWE, and (7) PO+10 mg/kg allopurinol (AP). Rats in groups (2-7) were injected intraperitoneally with 150 mg/kg PO prepared in 0.5% carboxymethyl cellulose (CMC) with 0.1 M sodium acetate (pH 5.0) to induce hyperuricemia, while the normal control (1) rats were treated with 0.5% CMC with 0.1 M sodium acetate. SMWW, SMWE, and AP were dispersed in 0.5% CMC and administered by oral gavage, 1 h prior to PO injection. 

### 4.5. Analysis of Uric Acid in Serum

Blood samples were collected via cardiac puncture, under anesthesia, 2 h after PO treatment. Serum was obtained by centrifugation at 3000× *g* for 10 min at 4 °C, after allowing the blood samples to clot for 2 h, at room temperature. The separated serum uric acid levels were determined, using an enzymatic-colorimetric method, using commercial assay kits (Biovision, Milpitas, CA, USA) according to manufacturer’s protocols.

### 4.6. Induction of Gouty Arthritis with MSU Crystals in Mice 

MSU was synthesized as previously described [44]. After acclimation, C57BL6 male mice (8 weeks old, 20-22g body weight) were divided into the following eight groups (*n* = 5/group): (1) normal controls, (2) MSU-crystal-treated, (3) MSU+100 mg/kg SMWW, (4) MSU+200 mg/kg SMWW, (5) MSU+50 mg/kg SMWE, (6) MSU+100 mg/kg SMWE, (7) MSU+200 mg/kg SMWE, and (8) MSU+1 mg/kg colchicine (Col). The right hind paw of each mouse in groups (2–8) was injected intradermally with MSU crystal suspension (4 mg/50 μL) in PBS with 0.5% Tween 80, while the normal control (1) mice were treated with PBS with 0.5% Tween 80. SMWW, SMWE, and Col were dispersed in 0.5% CMC and administered by oral gavage, 1 h before the MSU crystal injection, and then once daily, for 3 days. The experimental design is shown in Figure 3A.

### 4.7. Assessment of Inflammatory Paw Swelling and Pain

Inflammatory paw swelling was quantified by measuring the thickness of the MSU-injected paw, using a Vernier scale, 3 days after the induction of MSU. The change of thickness (mm) was calculated as follows: Change of thickness (mm) = MSU-treated paw thickness - normal control paw thickness [45]. The pain was measured by right and left hind-limb weight distribution, using a dynamic weight-bearing device (Bioseb, Boulogne, France), which was developed to measure the weight borne by each limb in freely moving animals [44,46]. The mice were placed in a small Plexiglas chamber (11.0 × 19.7 × 11.0 cm) with a floor sensor containing pressure transducer, for 2 minutes, and the analyzer recorded the average weight in grams, for each limb put on the floor. All movements were filmed and validated according to the position of the mouse on the device, and the results were analyzed for the weight of the paw, which touches the floor in grams [47]. The relative right/left hind paws weight-bearing distribution was calculated by using the following equation: (weight on right hind limb / weight on left hind limb) × 100.

### 4.8. Measurement of Inflammatory Cytokines and Mediators

The levels of IL-1β, IL-6, TNF-α, and myeloperoxidase (MPO) were measured by using ELISA kits from R&D Systems (Minneapolis, MN, USA) and MyBioSource (San Diego, CA, USA) according to the manufacturers’ protocols.

### 4.9. Statistical Analysis

The results were expressed as the mean ± standard error of the mean (SEM) and analyzed, using a one-way analysis of variance (ANOVA), followed by Dunnett’s tests for multiple comparisons or unpaired Student’s *t*-tests for two-group comparisons. Normality was performed by using Shapiro–Wilk’s test. All analyses were performed, using Prism 7.0 (GraphPad Software, San Diego, CA, USA), and *p*-values < 0.05 were considered significant.

## 5. Conclusions

In conclusion, this study demonstrated that SMWW and SMWE equally reduced serum uric acid levels in PO-induced hyperuricemic rats. However, in a gouty arthritis animal model, SMWE more efficiently downregulated MSU-crystal-induced swelling and pain, and it exerted anti-inflammatory effects by suppressing proinflammatory cytokines (IL-1β, TNF-α, and IL-6) and MPO activity. Moreover, berberine was found to be one of the most differentially abundant main active ingredients between SMWW and SMWE, while atractylenolide III was identified only in SMWE, both of which are known to elicit anti-inflammatory effects. These observations show that 30% ethanol is an efficient solvent for SMW extraction with anti-gouty arthritis efficacy at the concentrations reduced compared with water extracts. Further studies should be conducted to determine whether SMWE has similar efficacy in clinical trials at lower doses than SMWW. 

## Data Availability

The data presented in this study is contained within the article.

## Abbreviations

ADAMTSs	aggrecanases
AP	allopurinol
CMC	carboxymethyl cellulose
Col	colchicine
HPLC	high-performance liquid chromatography
IL-1β	interleukin-1β
IL-6	interleukin-6
iNOS	inducible nitric oxide synthase
JAK–STAT	Janus kinase–signal transducer and activator of transcription
LPS	lipopolysaccharide
MAPK	mitogen-activated protein kinase
MMPs	matrix metalloproteinases
MPO	myeloperoxidase
MSU	monosodium urate
NO	nitric oxide
NOD	nucleotide binding and oligomerization domain
PO	potassium oxonate
PGE2	prostaglandin E2
SMW	Sam-Myo-Whan
SMWE	SMW extracted with ethanol
SMWW	SMW extracted with water
TIMPs	tissue inhibitors of metalloproteinases
TNF-α	tumor necrosis factor-alpha
XOD	xanthine oxidase
